# Supplementary material for: Training certified detectives to track down the intrinsic shortcuts in COVID-19 chest x-ray data sets
Source: Sci Rep. 2023 Aug 4;13:12690. doi: 10.1038/s41598-023-39855-3 (PMC10403557; doi:10.1038/s41598-023-39855-3)
Supplement: Supplementary file 1 — Supplementary Information. [file 41598_2023_39855_MOESM1_ESM.pdf]

Training certified detectives to track down the intrinsic shortcuts in COVID-19 chest x-ray data sets

Ran Zhang[1*], Dalton Griner[1*], John W. Garrett[2,1], Zhihua Qi[3], and Guang-Hong Chen[1,2]

1. Department of Medical Physics, School of Medicine and Public Health, the University of Wisconsin in Madison, Madison, WI 53705

2. Department of Radiology, School of Medicine and Public Health, the University of Wisconsin in Madison, Madison, WI 53792

3. Department of Radiology, Henry Ford Health, Detroit, MI 48202

*Co-first authors

Address correspondence to:

Guang-Hong Chen, Ph.D.,

Department of Medical Physics and Department of Radiology,

School of Medicine and Public Health,

University of Wisconsin in Madison, Madison, WI 53705,

Email: gchen7@wisc.edu

Supplementary Information

Appendix A1. Technical details on adjusting ADA(C) and ADA(S) of the CXR

Technical details for adjusting these image attributes are elaborated in Figure A1. Note that our goal is to introduce realistic shortcuts which mimic the variations in the image generation pipeline shown in Figure 1. In other words, no drastic changes should be observed in the overall image appearance. For this purpose, the range of the image sharpness factor, s, was set to [-1.0,1.0]; the range of the image contrast factor, c, was set to [0.015, 0.020]. For each CXR, α and c are uniformly sampled from the given range. As shown in Figure A2, ADA(C) and ADA(S) adjusted images with the above parameters only introduce subtle changes to the original image.

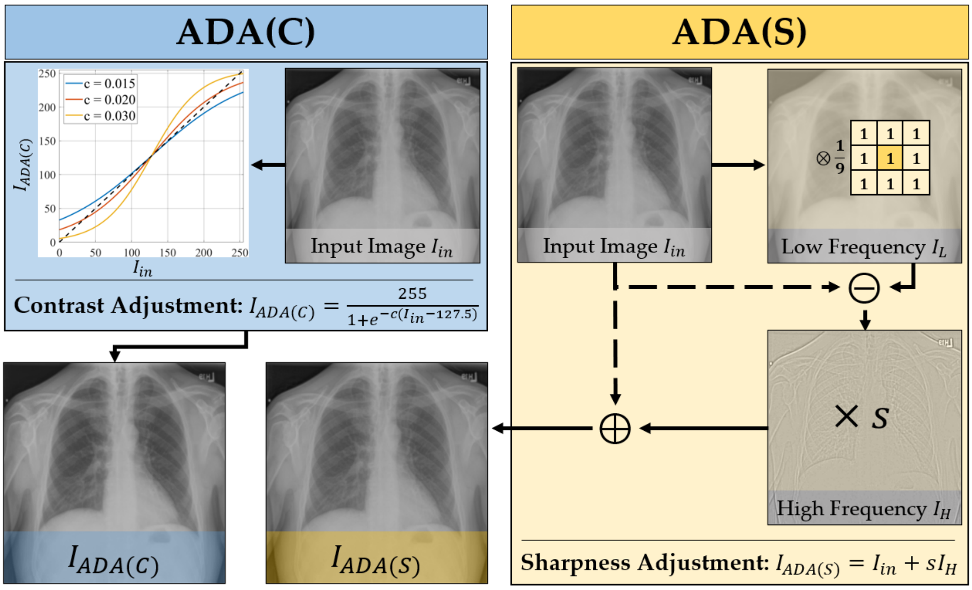

Figure A1. Methods for adjusting the image sharpness and contrast.

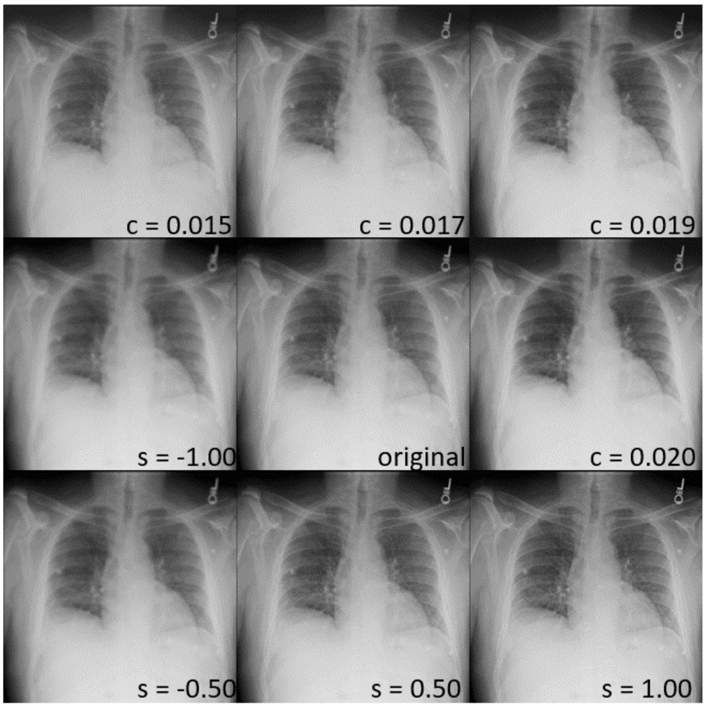

Figure A2. Examples of ADA(C) and ADA(S) adjusted images.

Appendix A2. Details of model training

The original CXR DICOM images are resized to 224 × 244 using bilinear interpolation and converted to 8-bit grayscale for all models used in this work. A three-stage transfer learning was applied to all model training. In stage 1), all models are initialized with ImageNet pre-trained weights; in Stage 2), a classifier replaced the original classifier with 1000-class output with 14-class output. The model was then trained using the NIH chest x-ray dataset with 14 disease labels; Stage 3), the final stage to reduce the classifier output to binary for the final training using COVID-19 chest x-ray datasets. Binary Cross-Entropy loss with the ADAM optimizer was used. The initial learning rate for all models is 1e-4. An 80%-20% random partition was used to generate training and validation sets. Data augmentation techniques were used for all model training, including random rotation (30° range) and horizontal flipping. The model with the lowest loss on the validation set was saved. An ensemble training strategy was implemented: Five individual models were trained, corresponding to different training-validation partitions for each dataset. The final prediction for an image is calculated as the quadratic mean of the five trained networks.

Appendix A3.

Table A1. Shortcut detectives trained using other model architectures

	**COVIDx**	**RoentGen -MIMIC**	**UW**	**BIMCV**	**MIDRC**
			VGG		
ADA(S) shortcut	**0.68 [0.68,0.69]**	**0.01 [0.01,0.01]**	0.47 [0.45,0.49]	0.50 [0.48,0.51]	0.44 [0.43,0.45]
ADA(C) shortcut	**0.74 [0.74,0.75]**	**0.05 [0.05, 0.06]**	0.49 [0.47,0.51]	0.56 [0.55,0.57]	0.49 [0.49,0.50]
			EfficientNet		
ADA(S) shortcut	**0.86 [0.85,0.86]**	**0.00 [0.00,0.00]**	0.49 [0.49,0.50]	0.48 [0.47,0.50]	0.37 [0.36,0.38]
ADA(C) shortcut	**0.78 [0.78,0.79]**	**0.06 [0.05, 0.07]**	0.48 [0.46,0.50]	0.54 [0.51,0.57]	0.52 [0.51,0.54]
			Swin Transformer		
ADA(S) shortcut	**0.71 [0.70,0.72]**	0.39 [0.37,0.42]	0.47 [0.46,0.49]	0.47 [0.46,0.48]	0.48 [0.47,0.48]
ADA(C) shortcut	**0.80 [0.80,0.81]**	**0.08 [0.07, 0.10]**	0.52 [0.50,0.53]	0.58 [0.57,0.59]	0.49 [0.48,0.49]
			ConvNext		
ADA(S) shortcut	**0.77 [0.77,0.78]**	**0.02 [0.01,0.02]**	0.53 [0.51,0.55]	0.49 [0.47,0.50]	0.38 [0.37,0.39]
ADA(C) shortcut	**0.75 [0.74,0.75]**	**0.07 [0.06, 0.08]**	0.49 [0.47,0.51]	0.55 [0.54,0.56]	0.52 [0.51,0.53]
